# Molecular Detection and Phylogenetic Analysis of *Anaplasma phagocytophilum* and Related Strains in Cattle from Henan, China

**DOI:** 10.3390/vetsci12030252

**Published:** 2025-03-06

**Authors:** Yaqun Yan, Yongli Wang, Yanyan Cui, Jin Wang, Shuhua Fan, Changshen Ning

**Affiliations:** 1 College of Life Science and Agronomy, Zhoukou Normal University, Zhoukou 466000, China; yanyq2022@zknu.edu.cn (Y.Y.); wangyl@zknu.edu.cn (Y.W.); wangjin7766@126.com (J.W.); 2 Field Observation and Research Station of Green Agriculture in Dancheng County, Dancheng 477150, China; 3 School of Biotechnology and Food, Shangqiu Normal University, Shangqiu 476000, China; yanyancui210116@163.com; 4 College of Veterinary Medicine, Henan Agricultural University, Zhengzhou 450046, China

**Keywords:** *Anaplasma phagocytophilum*, *A. phagocytophilum*-like 1 and 2, cattle, PCR-RFLP, phylogeny

## Abstract

*Anaplasma phagocytophilum* is a zoonotic pathogen transmitted by arthropod vectors. The pathogen infects various vertebrate hosts, causing mild to severe illness. Molecular studies have demonstrated that *A*. *phagocytophilum* exhibits a high level of genetic diversity, with two *A. phagocytophilum*-related variants identified in several countries. This study represents the first application of PCR amplification and restriction fragment length polymorphism (PCR-RFLP) in conjunction with DNA sequencing to investigate the frequency and phylogenetic relationships of *A. phagocytophilum* and its related strains in cattle from China. A total of 662 bovine blood samples were collected from diverse regions within Henan Province, China, and pathogen DNA was detected in 75 samples, comprising 11.33% of the total. PCR-RFLP analysis identified three strains with frequency rates of 2.87% (19/662) for *A. phagocytophilum*, 11.33% (75/662) for *A. phagocytophilum*-like 1, and 3.22% (22/662) for *A. phagocytophilum*-like 2. Additionally, co-infections involving *A. phagocytophilum* and *A. phagocytophilum*-like 1 were observed as well as between *A. phagocytophilum*-like 1 and *A. phagocytophilum*-like 2. *Anaplasma phagocytophilum*-like strains 1 and 2 should be considered when diagnosing bovine anaplasmosis. Despite recent molecular studies of *A. phagocytophilum*-related strains, there remains a shortage of data concerning vector capability, the epidemiology of the disease, clinical signs, and genetic diversity of the pathogens. Thus, large-scale investigations involving animals and tick vectors are necessary to obtain more detailed information concerning the etiology of anaplasmosis.

## Simple Summary

*Anaplasma phagocytophilum* exhibits significant genetic diversity, and studies have identified several variants of *A. phagocytophilum* across various geographic regions. In this investigation, 662 blood samples from cattle in Henan Province, China, were examined for *A. phagocytophilum* and related strains. The overall infection rate was 11.33%, with some cattle carrying two strains simultaneously. This study highlights the importance of including *A. phagocytophilum*-like strains in the diagnosis of anaplasmosis in cattle. Despite existing research on these bacteria, further large-scale studies are necessary to enhance our understanding of their dissemination, clinical manifestations in animals, and genetic variations.

## 1. Introduction

The Gram-negative bacterium *Anaplasma phagocytophilum* is transmitted by ticks belonging to the family Ixodidae [1]. This pathogen is transmitted to mammalian hosts in natural habitats by ixodid ticks, mainly from the *Ixodes* genus, such as *I. scapularis*, *I. pacificus*, *I. ricinus*, and *I. persulcatus*. In addition, it was also detected in ticks from other genera: *Amblyomma*, *Dermacentor, Haemaphysalis*, and *Rhipicephalus* [2,3]. *Anaplasma phagocytophilum* is a widely distributed zoonotic pathogen with a broad host range. The diseases related to *A. phagocytophilum* represent significant worldwide veterinary and public health concerns [4]. The pathogen invades the body and replicates within neutrophil granulocytes, resulting in febrile granulocytic anaplasmosis in a wide variety of hosts, including humans and domestic animals such as cattle, dogs, sheep, goats, horses, and cats [4,5]. In cattle, sheep, and goats, the disease is specifically referred to as tick-borne fever (TBF) [6]. TBF is characterized by high fever, lethargy, coughing, and anorexia [4]. In cattle, TBF has significant economic implications due to reduced milk production, reproductive issues such as spontaneous abortion, and immunosuppression that increases the susceptibility to secondary infections [7]. Leukopenia, thrombopenia, and anemia are typical laboratory findings [8]. *Anaplasma phagocytophilum* is one of the two most relevant pathogens causing bovine anaplasmosis, a globally prevalent tick-borne disease that poses a serious threat to the cattle industry [9,10,11].

An increased research focus on *A. phagocytophilum* has led to a deeper understanding of the genetic diversity of the pathogen. Recently, two novel *A. phagocytophilum*-related strains (*Anaplasma* spp. from Japan and China) have been classified as *A. phagocytophilum*-like 1 and *A. phagocytophilum*-like 2, respectively. Phylogenetic analyses of nucleotide sequences from the *16S rRNA*, citrate synthase (*gltA*), and heat-shock operon (*groEL*) genes form the basis of this classification [12,13,14,15,16,17]. Recent studies have demonstrated that SSAP2F and SSAP2R primers are specific to *A. phagocytophilum* and can effectively amplify the various strains [3,14,17,18,19,20]. To distinguish the strains and identify the co-infection between *A. phagocytophilum* and the two related strains, the restriction enzymes *XcmI* and *BsaI* were used to digest nested PCR amplicons of the *16S rRNA* gene using the primers mentioned [15]. Digestion of the amplicons of *A. phagocytophilum* with *XcmI* produced two RFLP fragments measuring 344 and 297 bp. In contrast, the corresponding PCR products of two novel *A. phagocytophilum*-related strains were not cleaved by the same enzyme. Using the restriction enzyme *BsaI* to degrade *16S rRNA* amplicons allowed for the separation of two novel *A. phagocytophilum*-related strains. *Anaplasma phagocytophilum*-like 1 remained undigested, whereas the amplified products of *A. phagocytophilum*-like 2 could be separated into two fragments measuring 422 (or 423) and 219 bp [15]. In addition, PCR-RFLP and DNA sequencing have identified two novel *A. phagocytophilum*-related strains in various hosts, including ruminants, small mammals, and diverse tick species. Specifically, *A. phagocytophilum*-like 1 was discovered in deer, cattle, *Ixodes* ticks, *Haemaphysalis longicornis* [21,22], and *H. megaspinosa* from Japan [23], in cattle from South Korea [24], Xinjiang in China [25], and Kyrgyzstan [18], in *Rattus rattus* in Tunisia [26], in sheep, goats, and water buffalo in Turkey [20,27,28], and in *H. bispinosa* in Thailand [29]. *Anaplasma phagocytophilum*-like 2 has been detected in *Hyalomma asiaticum*, as well as in sheep and goats in China [13,30]. The two new strains related to *A. phagocytophilum* have been reported in Turkish cattle, sheep, and goats from the Mediterranean [16,17]. Despite the substantial body of literature on the epidemiology of *A. phagocytophilum*, there is a notable lack of data regarding the global prevalence and distribution of *A. phagocytophilum*-related strains [31].

While there have been reports of *A. phagocytophilum* infecting cattle, to date, investigations specifically targeted toward *A. phagocytophilum*-like strains in cattle from China remain limited [25,30,32,33]. Some studies suggest that the pathogenicity of the three isolates is inconsistent [15,16]. Additionally, it is unclear whether co-infections involving both *A. phagocytophilum* and related strains occur simultaneously. Thus, comprehending the prevalence and distribution of different isolates is crucial for the effective prevention and control of bovine anaplasmosis. Henan province, which is approximately 167,000 km^2^, has the country’s sixth-highest number of cattle. As of 2021, Henan province contained approximately 398,000 heads [34]. Therefore, this study presents an initial comprehensive evaluation of *A. phagocytophilum* and *A. phagocytophilum*-like strains in cattle from Henan, China, using PCR-RFLP targeting the *16S rRNA* gene. The frequency, co-infection rates, and molecular characteristics of the pathogen were determined.

## 2. Materials and Methods

### 2.1. Sample Collection and DNA Extraction

**Figure 1:**
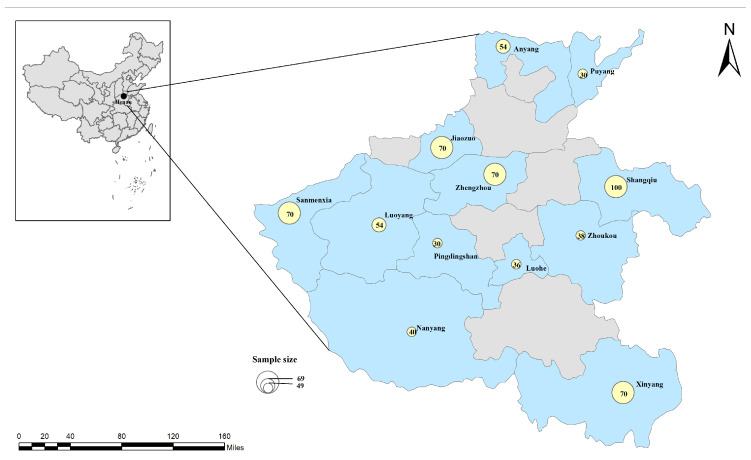
Sampling locations in China. The figure was designed with ArcGIS 10.8 using a vector diagram from Natural Earth (http://www.naturalearthdata.com, accessed on 31 October 2024) as a base. These yellow circles represent the sample sizes collected in each region.

A total of 662 blood samples were collected randomly from cattle in 12 cities of Henan Province, central China (latitude 31°23′–36°22′ N, longitude 110°21′–116°39′ E) during the period from August to October 2022. Blood (approximately 2 mL) from the caudal vein of each animal was collected in 5 mL sterile EDTA tubes. The sampling locations are depicted in Figure 1.

DNA was extracted from 250 μL aliquots of EDTA-treated blood samples utilizing a DNeasy Blood and Tissue Kit (OMEGA, Norcross, GA, USA). Each sample was processed individually following the manufacturer’s instructions. A NanoDrop-2000 spectrophotometer was used to evaluate the concentration and quality of the extracted DNA. Samples exhibiting a minimum DNA concentration of 20 ng/μL were deemed suitable for subsequent PCR analysis. The extracted DNA was preserved at −20 °C until further use.

### 2.2. Detection of Strains by PCR and Restriction Fragment Length Polymorphism (RFLP)

To detect the presence of *A. phagocytophilum* and *A. phagocytophilum*-like strains, nested PCR targeting the *16S rRNA* gene was performed following previously published protocols (Table 1) [23,35]. Several studies have demonstrated that the inner primers (SSAP2f/SSAP2r) can specifically detect *A. phagocytophilum* and *A. phagocytophilum*-like strains, yielding a target band of 641–642 bp [12,14,15,16]. The restriction enzymes *XcmI* and *BsaI* (New England BioLabs, Hitchin, UK) were used to digest nested PCR amplicons.

The enzyme digestion reaction analysis was performed under the following conditions. The amounts of PCR amplicons, restriction enzymes, buffer, and ddH2O comprising the enzyme digestion reaction mixture were 10, 1, 2.5, and 11.5 µL, respectively. The reaction mixture was initially incubated at 37 °C for 1 h, followed by an incubation at 65 °C for 20 min to facilitate *XcmI* digestion. For *BsaI*, the reaction was carried out at 37 °C for 5–15 min, followed by incubation at 80 °C for 20 min [15].

Positive samples identified by the *16S rRNA* gene were further analyzed to characterize the different strains according to the methods outlined in previous studies [16,36,37]. The oligonucleotide primers and amplification conditions are specifically designed to amplify *A. phagocytophilum* and two *A. phagocytophilum*-related strains are presented in Table 1.

### 2.3. DNA Cloning

The presence of co-infection with different strains makes it challenging to obtain reliable gene sequences directly from mixed infection samples. To accurately determine the sequences of individual isolates, positive PCR products derived from co-infected samples identified by RFLP were purified using an agarose gel extraction kit (TIANGEN, Beijing, China) according to the manufacturer’s instructions. The purified products were ligated into the pMD-18T vector (TaKaRa, Dalian, China) using T4 DNA ligase, following the standard protocol. The ligation products were transformed into *Escherichia coli* DH5α competent cells (Zoman, Beijing, China). Transformed cells were plated onto LB agar plates supplemented with ampicillin (100 µg/mL), X-Gal, and IPTG for blue-white screening. After incubation at 37 °C for 12–16 h, white colonies were selected and screened by colony PCR to confirm the presence of the target insert [38]. Positive clones were further verified by Sanger sequencing. Simultaneously, the PCR products of the positive clones were digested with two restriction enzymes (*XcmI* and *BsaI*) to facilitate strain identification.

### 2.4. Analysis of Sequencing and Phylogenetics

Sequencing was carried out by Sangon Biotech, Shanghai, China, for all PCR amplicons and selected positive clones. The accuracy of sequences was verified by bidirectional sequencing. Additionally, the sequences obtained were identified and analyzed through a BLASTn search against the GenBank database and aligned using ClustalW 2.0.10.

Phylogenetic analyses were performed on the *16S rRNA* and *groEL* genes from *A. phagocytophilum* and related strains identified using the optimal evolutionary model in MEGA 11.0 via the neighbor-joining method. DNA reference sequences for other *Anaplasma* species were downloaded from GenBank. The confidence values for each branch of the resulting tree were calculated using bootstrap analysis with 1000 replicates.

### 2.5. Statistical Analysis

The chi-square test was used to analyze the differences in the frequency of *A. phagocytophilum* and *A. phagocytophilum*-like strains among groups based on some factors, including sex, age, and feeding habits, using SPSS 27.0 software (SPSS Inc., Chicago, IL, USA). Statistically significant differences were identified with a *p*-value < 0.05, and 95% confidence intervals for each estimate were calculated.

### 2.6. Accession Numbers for Nucleotide Sequences

All consensus sequences from the present research were deposited in GenBank. The *16S rRNA* gene sequences of *A. phagocytophilum* and *A. phagocytophilum*-like strains have the following GenBank accession numbers: OL884352 and OL884353 for *A. phagocytophilum*, OL884219 and OL884220 for *A. phagocytophilum*-like 1, and OL884226 and OL884227 for *A. phagocytophilum*-like 2. The GenBank accession numbers for the *groEL* gene sequences are OL989885 for *A. phagocytophilum*-like 1 and OL989886 for *A. phagocytophilum*-like 2.

## 3. Results

### 3.1. Anaplasma spp. Frequency

**Figure 2:**
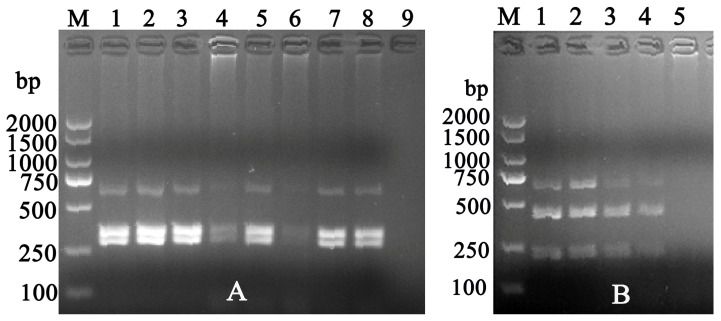
Results of partial DNA analysis with the restriction enzymes *XcmI* (**A**) and *BsaI* (**B**). Figure 2A: Line M: DNA 2000 bp Marker; lines 1–8: PCR products after RFLP assay of *Anaplasma phagocytophilum* (344 and 297 bp) and *A. phagocytophilum*-like 1 (641–642 bp) coinfection; line 9: negative control. Figure 2B: Line M: DNA 2000 bp Marker; lines 1–4: PCR products after RFLP assay of *A. phagocytophilum*-like 2 (422 or 423 and 219 bp) and *A. phagocytophilum*-like 1 (641–642 bp) coinfection. 5: negative control.

For the *16S rRNA* sequence analysis, 75 out of 662 (11.33%) cattle samples were positive for *A. phagocytophilum* or related strains (Table 2). The presence of *A. phagocytophilum*, *A. phagocytophilum*-like 1, and *A. phagocytophilum*-like 2 in cattle was confirmed by *XcmI* and *BsaI* restriction enzyme digestion of the 16S PCR products. The corresponding frequency rates were 2.87% (19/662), 11.33% (75/662), and 3.32% (22/662), respectively. Co-infections involving *A. phagocytophilum* and *A. phagocytophilum*-like 1, as well as *A. phagocytophilum*-like 1 and *A. phagocytophilum*-like 2, were also observed, with positive rates of 2.87% (19/662) and 3.32% (22/662), respectively (Table 2). The positive PCR products were digested by restriction enzymes *XcmI* and *BsaI* in sequence. Figure 2 shows typical electrophoresis analysis results of the restriction enzyme digestion using *XcmI* and *BsaI* on *A. phagocytophilum* and *A. phagocytophilum*-like 2. After digestion of the PCR products with *XcmI*, three fragments of 641–642 bp, 344 bp, and 297 bp were obtained (Figure 2A). In contrast, the same PCR products were not digested by *BsaI*. These results indicated co-infection with *Anaplasma phagocytophilum* and *A. phagocytophilum*-like 1 in certain blood samples. Furthermore, using the same RFLP assay, co-infection between *A. phagocytophilum*-like 1 and *A. phagocytophilum*-like 2 was identified in some samples.

The present study investigated the frequency of *A. phagocytophilum*, *A. phagocytophilum*-like 1, and *A. phagocytophilum*-like 2 in cattle, considering the differences in sex, age, and feeding habits (Table 3). There were significant differences in the overall infection rates of *A. phagocytophilum* and related strains between male and female cattle (*p* < 0.01). However, there was no significant difference in the infection rate of *A. phagocytophilum*-like 2 (*p* > 0.05). Additionally, the overall infection rates decreased with age (0.01 < *p* < 0.05). Moreover, the infection rates of *A. phagocytophilum*, *A. phagocytophilum*-like 1, and *A. phagocytophilum*-like 2 were significantly higher in grazing cattle (13.41%, 54.88%, and 19.51%, respectively) compared to those fed in household settings (1.38%, 5.17%, and 1.03%, respectively; *p* < 0.01).

### 3.2. Molecular Characterization of Anaplasma spp. 16S rRNA Sequence Types

The PCR amplicons and selected clones of the *16S rRNA* gene were sequenced to confirm the results of the RFLP assay and detect genetic variants. Sequencing of the *16S rRNA* PCR amplicons and subsequent comparisons identified six distinct sequence types. These 16S rRNA sequence types were nearly identical (99.20–100%) to *A. phagocytophilum* or related isolates in the GenBank database, as revealed by BlastN comparisons.

**Figure 3:**
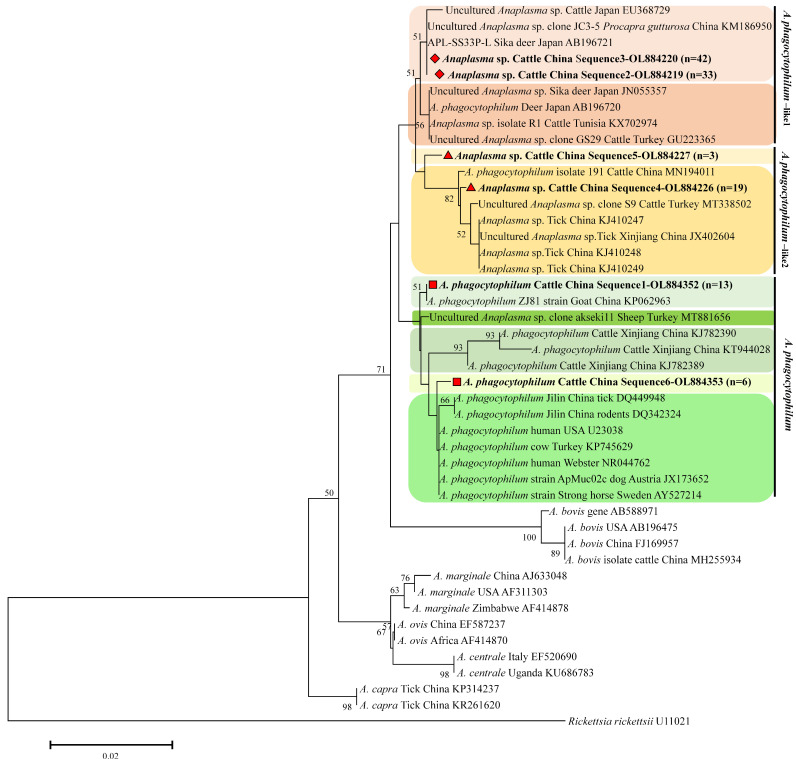
Phylogenetic tree of *Anaplasma* spp. based on the *16S rRNA* gene using the neighbor-joining method. The tree was constructed using the optimum evolutionary model and 1000 bootstrap replicates. The sequences for *A. phagocytophilum*, *A. phagocytophilum*-like 1, and *A. phagocytophilum*-like 2 in the present study are marked with red squares, red diamonds, and red triangles, respectively. *Rickettsia rickettsii* was used as an outgroup, and bootstrap values below 50% were omitted from the tree.

A phylogenetic tree was constructed based on the *16S rRNA* gene by aligning the six sequence types identified in this research with *Anaplasma* spp. strains from both ticks and animals, as well as representative *A. phagocytophilum* and two novel isolates (Figure 3). This approach was employed to validate the results obtained from the restriction enzyme digestion analysis by PCR-RFLP (Figure 2). Specifically, the genotypes (OL884352, *n* = 13 and OL884353, *n* = 6) identified in this study clustered within the *A. phagocytophilum* clade that included sequences from goats (KP062963), ticks (DQ449948), cattle (KT944028, KJ782389, KJ782390), and rodents (DQ342324) from China and sheep (MT881656) and cows (KP765429) from Turkey, as well as human (U23038, NR044762), dog (JX173652), and horse (AY527214) sequences. Two additional genotypes (OL884219, *n* = 33 and OL884219, *n* = 42) were placed within a clade that included other *A. phagocytophilum*-like 1 isolates from Japanese cattle (EU368729), deer (AB196720, AB196721, and JN055357), Chinese *Procapra gutturosa* (KM186950), and cattle from Tunisia and Turkey (KX702974, GU223365). In the phylogenetic tree, the sequences (OL884226, *n* = 19 and OL884227, *n* = 3) formed a distinct *A. phagocytophilum*-like 2 cluster, being closely related to sequences from ticks (KJ410247, KJ410248, KJ410249, and JX402604) and cattle (MN194011) from China and Turkey.

### 3.3. Molecular Characterization of Anaplasma spp. GroEL Sequence Types

Samples that tested positive for the 16S rRNA gene of *A. phagocytophilum*, *A. phagocytophilum*-like 1, and *A. phagocytophilum*-like 2 by RFLP were further analyzed using *groEL* nested or semi-nested PCR. Among these, 71 samples that tested positive for *A. phagocytophilum*-like 1 and 19 samples that tested positive for *A. phagocytophilum*-like 2 also yielded positive results for the *groEL* gene, confirming the findings from the *16S rRNA* analysis. However, no positive amplification of *groEL* was obtained from the specimens of *A. phagocytophilum*. Two distinct *groEL* sequence types were identified based on nucleotide alignments and comparative sequence analysis. The *groEL* sequences (GenBank accession nos. OL989885 and OL989886) showed 100% identity with those of *Anaplasma* spp. and *Candidatus A. boleense* (GenBank accession nos. KX388351 and KX987390, respectively).

**Figure 4:**
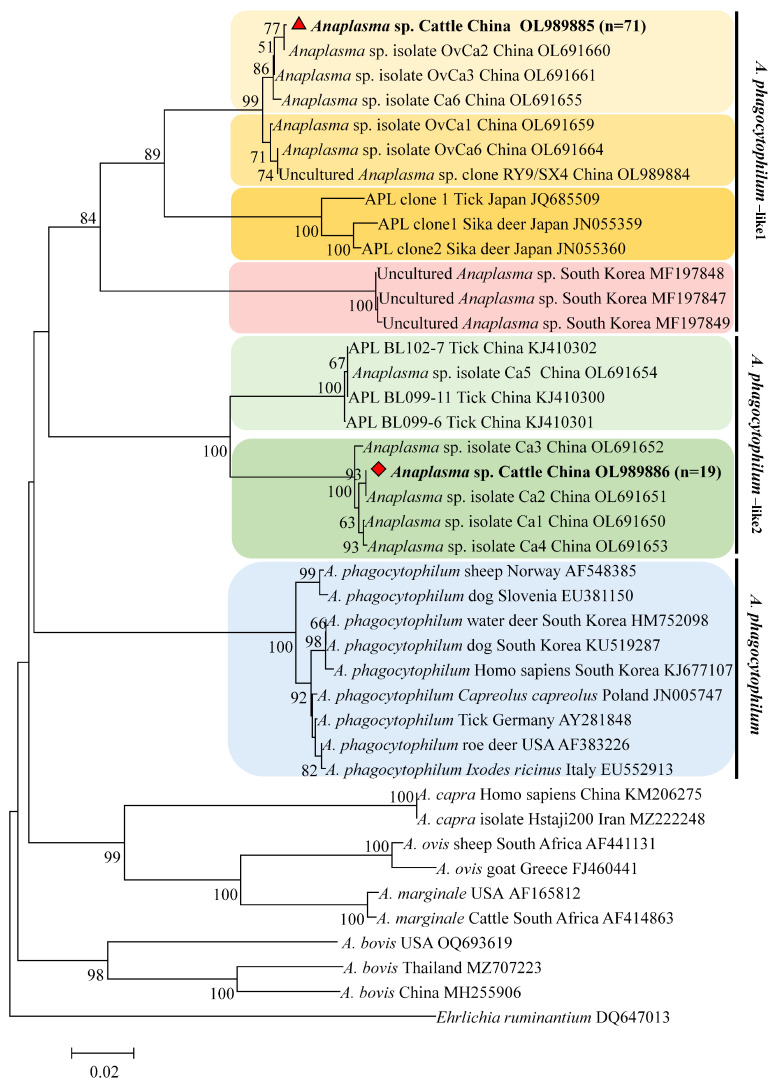
Phylogenetic tree of *Anaplasma* spp. based on the *groEL* gene using the neighbor-joining method. The tree was constructed using the optimum evolutionary model and 1000 bootstrap replicates. The sequences for *A. phagocytophilum*-like 1 and *A. phagocytophilum*-like 2 are marked with red triangles and red diamonds, respectively. *Ehrlichia ruminantium* was used as an outgroup, and bootstrap values below 50% were omitted from the tree.

Phylogenetic analysis of the *groEL* gene revealed that the two variants were closely related to *A. phagocytophilum* but were classified into distinct clades: one clustered with *A. phagocytophilum*-like 1 from sika deer, sheep, goats, and ticks, and the other clustered with *A. phagocytophilum*-like 2 from ticks and goats (Figure 4).

## 4. Discussion

In the present investigation, the overall frequency of *A. phagocytophilum* and related strains in cattle was 11.33% (75/662), a value that is higher than the frequency reported in cattle from Henan (3.49%, 3/86) [39], Chongqing (4.93%, 17/345) [33], and Xinjiang (6.40%, 8/125; 2.64%, 13/493) [25,30] as well as from another study conducted in China (10.43%, 12/115) [40]. However, this value was lower than the frequency observed in Iran (15.45%, 286/1851) [41] and Turkey (30.83%, 41/133) [11]. Previous studies in China based on evolutionary analyses have also identified *A. phagocytophilum* and two related strains in cattle, goats, and *Haemaphysalis longicornis* [25,42,43]. Nevertheless, it remained unclear whether *A. phagocytophilum* and *A. phagocytophilum*-like strains coexisted in cattle.

Blood samples were researched using SSAP2F and SSAP2R based on the *16S rRNA* gene, and after that, the *XcmI* and *BsaI* restriction enzymes were used to digest nested PCR amplicons to distinguish the strains and identify the co-infection between *A. phagocytophilum* and the two related strains in positive samples [15]. In the present study, we provide molecular evidence for the presence of *A. phagocytophilum*, *A. phagocytophilum*-like 1, and *A. phagocytophilum*-like 2 in cattle from Henan Province, China, based on PCR-RFLP analysis, marking the first report of these strains in the region. The positive rates of *A. phagocytophilum* (2.87%, 19/662) and *A. phagocytophilum*-like 1 (11.33%, 75/662) were higher than those reported from South Korea [24], Turkey [17], and Kyrgyzstan [44], where the frequency of *A. phagocytophilum* ranged from 2.14% (16/746) to 4.00% (8/200) and that of *A. phagocytophilum*-like 1 ranged from 3.20% (17/531) to 4.00%. The frequency of *A. phagocytophilum*-like 2 (3.32%, 22/662) was also higher than that reported in cattle from Turkey (1.50%, 3/200) [17]. Additionally, we detected *A. phagocytophilum* and related strains in the blood of cattle, irrespective of their feeding habits (grazing or household feeding). The feeding method was significantly associated with the positive rates. The frequency of these strains was significantly higher in grazing animals compared to that in household-fed animals (Table 2). This finding is consistent with previous studies that reported animals browsing outdoors were at higher risk than those raised indoors [25,45]. The increased likelihood of exposure to ticks in free-grazing conditions may explain the higher infection rates observed in grazing cattle. Thus, the difference in positive rates between feeding habits in this study is likely attributable to varying levels of tick exposure. Under grazing conditions, there is an increased incidence of tick bites, which may lead to a higher rate of *Anaplasma* spp. infection. Furthermore, the age and sex of animal hosts are regarded as significant risk factors. In this study, the rates of *A. phagocytophilum* and related strain infections were 17.13% and 9.95% in cattle younger than 2 years and between 2 and 5 years, respectively (0.01 < *p* < 0.05). However, significantly higher prevalence rates of anaplasmosis were recorded in older cattle than in younger ones in several studies [46,47]. None of the samples from cattle older than 5 years tested positive for *A. phagocytophilum* or related strains. This result might be attributed to the small sample size in this age group. Moreover, the positive rates of *A. phagocytophilum* and related strains were higher in males compared to females, with statistically significant differences. And previous data also supported our finding that sex had significant associations with *Anaplasma* spp. The finding is consistent with those of previous studies on *Anaplasma* spp. in cattle from China [33], South Korea [24], Tunisia [48], and Thailand [49] and in buffalo from Pakistan [50]. Another investigation found no significant association between genders and the prevalence of *Anaplasma* spp. in the univariate analysis [45].

The current study identified three distinct isolates based on PCR-RFLP analysis. Concurrent infections involving both *A. phagocytophilum* and *A. phagocytophilum*-like 1, as well as *A. phagocytophilum*-like 1 and *A. phagocytophilum*-like 2, were also detected (Table 2). Several studies have reported the presence of two isolates of *A*. *phagocytophilum* (*A. phagocytophilum* and *A. phagocytophilum*-like 2, or *A. phagocytophilum*-like 1 and *A. phagocytophilum*-like 2) in cattle [3,25]. Although *A. phagocytophilum* and its closely related strains have been identified in small ruminants, *A. phagocytophilum*-like 1 was particularly prevalent but appeared in only a limited number of samples along with *A. phagocytophilum* and *A. phagocytophilum*-like 2, as documented in previous studies [3,19,20]. The potential host specificity of these isolates remains uncertain. Furthermore, it is generally accepted that *A. phagocytophilum*-like 1 does not induce clinical manifestations, given its detection in clinically healthy animals [14,15,16,19,20]. However, some studies suggest that genetic variants of *A. phagocytophilum* may cause clinical symptoms in hosts [51,52]. On the other hand, there is currently no confirmed information on the zoonotic potential of two strains closely related to *A. phagocytophilum*. Consequently, further investigation into the pathogenicity of *A. phagocytophilum*-like 1 and -2 is warranted, as there are currently no reports linking these isolates to clinical manifestations in affected hosts. And we recommend screening individuals in *Anaplasma*-endemic areas for *A. phagocytophilum*-like 1 and 2 if they exhibit non-specific clinical symptoms during tick-active periods. This will provide valuable information on the zoonotic potential of these pathogens.

In recent years, DNA sequencing is used to confirm PCR results, perform phylogenetic analysis, evaluate genetic diversity, and identify new species [26,53,54,55]. In this study, it verified PCR and RFLP outcomes and assessed the genetic diversity of *A. phagocytophilum* and related strains. These *16S rRNA* sequence results obtained were agreed with those for *A. phagocytophilum* and related strains available in GenBank identified from different hosts, with 99.20–100% similarity. Several studies have shown significant genetic diversity among isolates of *A. phagocytophilum*. And in addition to the highly conserved *16S rRNA* gene, *groEL*, *msp4*, and *ankA* genes have been utilized to identify distinct variants circulating among animals [15,53,56,57,58]. However, contradictory results have been obtained depending on the locus used. For example, based on *ankA* gene phylogeny, isolates from roe deer and domestic ruminants (sheep and cattle) belonged to different clusters. In contrast, when examining the groEL locus, isolates from domestic ruminants (goats) belonged to the same cluster as those from roe deer [59,60]. Moreover, current markers cannot reveal the full genetic diversity of *A. phagocytophilum*. Multilocus sequence typing (MLST) methods were developed to at least partially solve these problems [61,62]. These methods have enhanced resolution power compared to single locus sequence typing in some previous studies [57,63]. Comprehensive molecular studies should be needed using multiple gene sequences to obtain more information about the genetic diversity of *A. phagocytophilum* and related strains.

The present findings notwithstanding, we acknowledge certain limitations in the present study, including the sample size and geographic scope. Moreover, while *A. phagocytophilum* is well-known to be zoonotic and transmitted by ticks [2], there is a lack of information regarding the potential role of ticks in the transmission of *A. phagocytophilum*-like variants. Additional studies with larger sample sizes and expanded geographic coverage are needed to better understand the frequency of *A. phagocytophilum* and related strains in cattle, as well as to identify the tick species that may serve as vectors for *A. phagocytophilum*-like variants.

## 5. Conclusions

In conclusion, this study provides the first molecular detection and phylogenetic analysis of *A. phagocytophilum* and related strains in cattle in China. The analysis demonstrated an overall frequency of 11.33%, as determined by the 16S rRNA gene in combination with RFLP assays. Given the capacity of *A. phagocytophilum* to cause severe infections, detecting infected or carrier cattle is critical for protecting human and animal health. Future research should consider *A. phagocytophilum*-like strains in the differential diagnosis of bovine anaplasmosis.

## Figures and Tables

**Table 1:** Specific oligonucleotide primers and PCR amplification conditions.

Target Gene	Isolates	Primer Name	Oligonucleotide Sequence (5′–3′)	Amplicon Size (bp)	Annealing	Reference
*16S rRNA*	AP and related variants	EE1	TCCTGGCTCAGAACGAACGCTGGCGGC	1430	55 °C	[35]
		EE2	GTCACTGACCCAACCTTAAATGGCTG			
		SSAP2f	GCTGAATGTGGGGATAATTTAT	641–642	55 °C	[23]
		SSAP2r	ATGGCTGCTTCCTTTCGGTTA			
*groEL*	AP	EphplgroEL-F	ATGGTATGCAGTTTGATCGC		55 °C	[36]
		EphplgroEL-R	TCTACTCTGTCTTTGCGTTC	642		
		EphgroEL-R	TTGAGTACAGCAACACCACCGGAA	573		
	AP-like 1	groEL-1F	TATAGCTAGCATAATTACCCAGAGC	339	53 °C	[37]
		groEL-1R	GGTTAGTTCTGCTTTCGATGC			
		groEL-2F	TTATGTCTATGCGCCGTG		51 °C	
		groEL-2R	CGGACCTTGCCACATTTT			
	AP-like 2	APHAGOVAR2GROEL_F	TACTCTAGAAGACGCGGTAG		55 °C	[16]
		APHAGOVAR2GROEL_R1	ACGAACATTCTTAGCAGTCC	792		
		APHAGOVAR2GROEL_R2	CTTCTATCACCAAATCCTGG			

AP: *A. phagocytophilum*; AP-like 1: *A. phagocytophilum*-like 1; AP-like 2: *A. phagocytophilum*-like 2.

**Table 2:** Origins of samples and the results of PCR and RFLP analyses.

Geographic Location	Tested Number	Positive (%)								Co-Infected (%)			
		16S rRNA+	95% CI ^a^	AP	95% CI ^a^	AP-like 1	95% CI ^a^	AP-like 2	95% CI ^a^	AP/AP-like 1	95% CI ^a^	AP-like 1/AP-like 2	95% CI ^a^
Luoyang	54	8 (14.81)	5.03–24.60	2 (3.70)	0–8.91	8 (14.81)	5.03–24.60	2 (3.70)	0–8.91	2 (3.70)	0–8.91	2 (3.70)	0–8.91
Luohe	36	2 (5.56)	0–13.42	0	—	2 (5.56)	0–13.42	0	—	0	—	0	—
Zhoukou	38	8 (21.05)	7.47–34.63	3 (7.89)	0–16.88	8 (21.05)	7.47–34.63	1 (2.63)	0–7.96	3 (7.89)	0–16.88	1 (2.63)	0–7.96
Anyang	54	19 (35.19)	22.03–48.34	6 (11.11)	2.45–19.77	19 (35.19)	22.03–48.34	5 (9.26)	1.27–17.25	6 (11.11)	2.45–19.77	5 (9.26)	1.27–17.25
Puyang	30	0	—	0	—	0	—	0	—	0	—	0	—
Xinyang	70	17 (24.29)	14.00–34.58	6 (8.57)	1.85–15.29	17 (24.29)	14.00–34.58	8 (11.43)	3.79–19.07	6 (8.57)	1.85–15.29	8 (11.43)	3.79–19.07
Shangqiu	100	5 (5.00)	0.65–9.35	0	—	5 (5.00)	0.65–9.35	0	—	0	—	0	—
Jiaozuo	70	7 (10.00)	2.80–17.20	2 (2.86)	0–6.86	7 (10.00)	2.80–17.20	5 (7.14)	0.96–13.33	2 (2.86)	0–6.86	5 (7.14)	0.96–13.33
Zhengzhou	70	0	—	0	—	0	—	0	—	0	—	0	—
Pingdingshan	30	0	—	0	—	0	—	0	—	0	—	0	—
Sanmenxia	70	2 (2.86)	0–6.86	0	—	2 (2.86)	0–6.86	1 (1.43)	0–4.28	—	—	1 (1.43)	0–4.28
Nanyang	40	7 (17.50)	5.19–29.81	0	—	7 (17.50)	5.19–29.81	0	—	0	—	0	—
Total	662	75 (11.33)	8.91–13.75	19 (2.87)	1.59–4.15	75 (11.33)	8.91–13.75	22 (3.32)	1.95–4.69	19 (2.87)	1.59–4.15	22 (3.32)	1.95–4.69

RFLP: Restriction Fragment Length Polymorphism; AP: *A. phagocytophilum*; AP-like 1: *A. phagocytophilum*-like 1; AP-like 2: *A. phagocytophilum*-like 2; ^a^ CI: Confidence interval.

**Table 3:** Detailed analysis of the differences in the frequency of *Anaplasma phagocytophilum* and related strains according to sex, age, and feeding habits.

Group		Tested	Positive (%)															
			16S rRNA+	95% CI ^a^	*p*-Value ^b^	OR	AP	95% CI ^a^	*p*-Value ^b^	OR	AP-like 1	95% CI ^a^	*p*-value ^b^	OR	AP-like 2	95% CI ^a^	*p*-Value ^b^	OR
Sex	Female	607	60 (9.88)	0.15–0.56	*p* < 0.01	0.29	15 (2.47)	0.10–1.01	0.01 < *p* < 0.05	0.32	60 (9.88)	0.15–0.56	*p* < 0.01	0.29	20 (3.29)	0.21–4.00	*p* > 0.05	0.90
	Male	55	15 (27.27)	—	—	1	4 (7.27)		—	1	15 (27.27)	—	—	1	2 (3.64)	—	—	1
Age	<2	181	31 (17.13)	1.14–3.07	0.01 < *p* < 0.05	1.87	10 (5.52)	1.12–7.04	0.01 < *p* < 0.05	2.81	31 (17.13)	1.14–3.07	0.01 < *p* < 0.05	1.87	10 (5.52)	0.89–4.94	*p* > 0.05	2.10
	2–5	442	44 (9.95)	—	—	1	9 (2.04)	—	—	1	44 (9.95)	—	—	1	12 (2.71)	—	—	1
	>5	39	0	—	0.01 < *p* < 0.05	—	0	—	*p* > 0.05	—	0	—	*p* > 0.05	0.9	0	—	*p* > 0.05	—
Feeding habits	Grazing	82	45 (54.88)	12.62–39.40	*p* < 0.01	22.30	11 (13.41)	4.3–28.46	*p* < 0.01	11.08	45 (54.88)	12.62–39.40	*p* < 0.01	22.30	16 (19.51)	8.77–61.32	*p* < 0.01	23.19
	Household	580	30 (5.17)	—	—	1	8 (1.38)	—	—	1	30 (5.17)	—	—	1	6 (1.03)	0–2.0	—	1

AP: *A. phagocytophilum*; AP-like 1: *A. phagocytophilum*-like 1; AP-like 2: *A. phagocytophilum*-like 2; ^a^ CI: Confidence interval; ^b^ Significant difference was observed (*p* < 0.05).

## Data Availability

The data presented in this study are available upon request from the corresponding author. The availability of the data are restricted to investigators based in academic institutions.
